# FusB Energizes Import across the Outer Membrane through Direct Interaction with Its Ferredoxin Substrate

**DOI:** 10.1128/mBio.02081-20

**Published:** 2020-10-27

**Authors:** Marta Wojnowska, Daniel Walker

**Affiliations:** a Institute of Infection, Immunity and Inflammation, College of Medical, Veterinary and Life Sciences, University of Glasgow, Glasgow, United Kingdom; University of Georgia

**Keywords:** *Pectobacterium*, TonB, ferredoxin, outer membrane, plant pathogens, plant-microbe interactions, protein transport

## Abstract

Phytopathogenic *Pectobacterium* spp. import ferredoxin into the periplasm for proteolytic processing and iron release via the ferredoxin uptake system. Although the ferredoxin receptor FusA and the processing protease FusC have been identified, the mechanistic basis of ferredoxin import is poorly understood. In this work, we demonstrate that protein translocation across the outer membrane is dependent on the TonB-like protein FusB. In contrast to the loss of FusC, loss of FusB or FusA abolishes ferredoxin transport to the periplasm, demonstrating that FusA and FusB work in concert to transport ferredoxin across the outer membrane. In addition to an interaction with the “TonB box” region of FusA, FusB also forms a complex with the ferredoxin substrate, with complex formation required for substrate transport. These data suggest that ferredoxin transport requires energy transduction from the cytoplasmic membrane via FusB both for removal of the FusA plug domain and for substrate translocation through the FusA barrel.

## Abstract (precis)

The ability to acquire iron is key to the ability of bacteria to cause infection. Plant-pathogenic *Pectobacterium* spp. are able to acquire iron from plants by transporting the iron-containing protein ferredoxin into the cell from proteolytic processing. In this work, we show that the TonB-like protein FusB plays a key role in transporting ferredoxin across the bacterial outer membrane by directly energizing its transport into the cell. The direct interaction of the TonB-like protein with substrate is unprecedented and explains the requirement for the system-specific TonB homologue in the ferredoxin uptake system. Since multiple genes encoding TonB-like proteins are commonly found in the genomes of Gram-negative bacteria, this may be a common mechanism for the uptake of atypical substrates via TonB-dependent receptors.

## INTRODUCTION

Gram-negative bacteria have evolved a number of strategies for the acquisition of iron and other nutrients in which “TonB-dependent” transporters (TBDTs) play a central role (1). In the case of siderophore-mediated iron acquisition, the iron-siderophore complex is imported into the cell, captured by a siderophore-specific periplasmic binding protein, and delivered to an ABC transporter for importation into the cytoplasm (2). For iron acquisition from large host proteins such as transferrin, the iron-containing protein is captured at the cell surface through TBDT binding and the iron stripped and subsequently transported through the lumen of the TBDT (3). In addition to the outer membrane (OM) receptor, whose lumen constitutes the translocation route, TBDT-mediated transport requires a complex of three proteins anchored in the inner membrane: TonB, ExbB, and ExbD (4, 5). The ExbBD-TonB complex enables the entry of the nutrient by removal of a force-labile portion of the plug domain, which obstructs the receptor lumen (6). ExbB and ExbD are related to the flagellar motor proteins and harness proton motive force (PMF) to energize the transport process.

In addition to the uptake of iron siderophores and other metal chelating compounds such as vitamin B12, TBDTs also transport complex carbohydrates and simple sugars (7). A recent study also described the role of a TonB-dependent receptor in protein export, suggesting that TonB-dependent receptors are highly adaptable to the transport of diverse substrates across the OM (8). The flexibility in the range of substrates that are amenable to transport by TBDTs is exploited by protein antibiotics such as colicins and pyocins that use TBDTs as their primary cell surface receptor and translocator (9). As with the uptake of nutrients, translocation of colicins and pyocins via TBDTs is PMF dependent, although the periplasm-spanning protein TonB is required in such cases both to remove the force-labile region of the TBDT-plug domain and subsequently to energize protein translocation across the OM (10). Protein translocation occurs by direct interaction with an N-terminal intrinsically unstructured region of the toxin that, similarly to the TBDTs, carries a TonB-binding motif (10).

We recently demonstrated that TBDT-mediated iron acquisition from the iron-sulfur cluster containing protein ferredoxin represents an unprecedented example of protein translocation into the bacterial cell for nutrient acquisition (11). Ferredoxin binding at the cell surface is mediated by the TBDT FusA protein, and, following transport of intact ferredoxin into the periplasm, the substrate is subjected to proteolytic processing by the M16 protease FusC (11, 12). Cleavage by FusC results in release of the iron-sulfur cluster and is required for effective iron acquisition from ferredoxin by *Pectobacterium*. Together with the genes encoding FusA and FusC, the Fus operon contains two additional genes, with *fusB* encoding a TonB homologue and *fusD* a putative ABC transporter (12). Interestingly, the M-type pectocins M1 and M2, which we have previously described, parasitize the ferredoxin uptake system through an N-terminal ferredoxin domain that is highly homologous to plant ferredoxin domains (13, 14).

More recently, the X-ray structure of FusC bound to ferredoxin has been reported, showing that substrate recognition occurs at a site distant from the active site (15). Furthermore, only parts of the ferredoxin molecule are visible in the structure, implying that the bound substrate is largely present in an unstructured form. On the basis of these data, it was suggested that ferredoxin transport occurs by means of a Brownian ratchet mechanism in which FusC acts as a periplasmic anchor to facilitate translocation of ferredoxin across the OM via the lumen of FusA (12, 15). Similar mechanisms have been postulated to account for mitochondrial protein uptake, whereby cytoplasmically synthesized proteins are translocated via the TOM and TIM23 complexes (16, 17). As such, this would represent a hitherto-unexpected evolutionary link between mitochondrial and plastid protein import and bacterial protein import via the Fus uptake system (FUS) and other postulated protein uptake systems.

In this work, we show that FusC does not facilitate ferredoxin import and that, like the import of other TBDT substrates, ferredoxin uptake is PMF dependent. Instead, we show that the TonB homologue encoded within the *fus* operon, FusB, is required for ferredoxin import and that the mechanism of ferredoxin import involves a direct interaction between FusB and the ferredoxin substrate. The evidence pointing to a direct interaction of the TonB-like protein with the substrate is unprecedented and explains the requirement for the system-specific TonB homologue in the Fus system. Our data also show that, in addition to the direct interaction with the substrate, FusB fulfils another role—similarly to other TonB proteins—in interacting with the “TonB box” of FusA for plug displacement. Since multiple genes encoding TonB-like proteins are commonly found in the genomes of Gram-negative bacteria, this may be a common mechanism for the uptake of atypical substrates via TonB-dependent receptors.

## RESULTS

### Ferredoxin import is independent of FusC but requires proton motive force.

We previously showed that FusC is a highly specific protease that targets plant ferredoxin to release iron from this host protein in the periplasm of *Pectobacterium* spp. (11). However, it has also been suggested that FusC plays an additional role in iron acquisition through a direct involvement in ferredoxin transport across the outer membrane by means of a Brownian ratchet mechanism, specifically acting as a periplasmic anchor (15). Our own previous work suggests that if FusC does play a role in ferredoxin import, this role is not essential since the accumulation of *Arabidopsis* ferredoxin can still be observed in a Pectobacterium carotovorum LMG2410 (*Pc*LMG2410) strain lacking FusC (11). However, using *Arabidopsis* ferredoxin (Fer_Ara_), it is not possible to directly compare the rates and extents of ferredoxin uptake between wild-type (WT) and *ΔfusC* P. carotovorum since Fer_Ara_ is cleaved by FusC upon importation into the periplasm in the WT strain; hence, on the basis of these data, we could not rule out a possible role for FusC in ferredoxin import.

**FIG 1:**
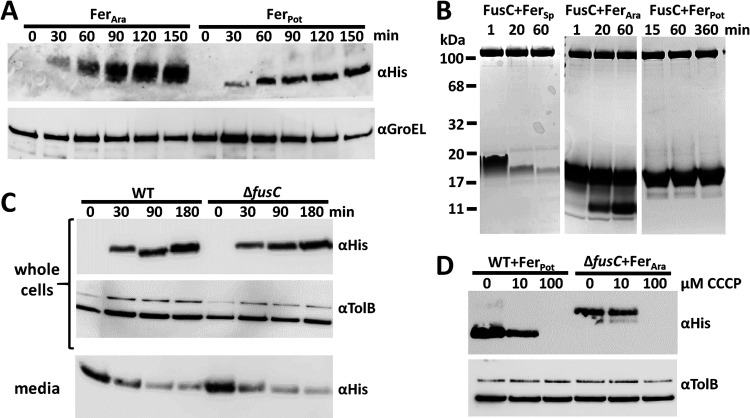
Import of ferredoxin is FusC independent and requires proton motive force. (A) Uptake of His-tagged Fer_Ara_ and Fer_Pot_ by Δ*fusC* cells over time determined by immunoblotting of whole-cell extracts. GroEL served as the loading control. (B) FusC-mediated cleavage assays of plant ferredoxins. Comparable cleavage rates were observed in >5 independent experiments. (C) Comparison of levels of Fer_Pot_ uptake by wild-type (WT) and Δ*fusC* cells, with TolB as the loading control. The bottom panel shows the concomitant depletion of ferredoxin from the media. (D) Ferredoxin import assays in the presence of protonophore CCCP. Fer_Pot_ was used as a reporter in wild-type cells, while Fer_Ara_ was used in Δ*fusC* cells. TolB served as the loading control.

To test the hypothesis that FusC facilitates translocation of ferredoxin to the periplasm, we compared the levels of uptake of potato ferredoxin (Fer_Pot_) in wild-type and *ΔfusC Pc*LMG2410. Fer_Pot_ was used as we had observed that although, similarly to Fer_Ara_, it can be transported into cells (Fig. 1a), unlike Fer_Ara_ and spinach ferredoxin (Fer_Sp_), both of which support robust growth of *Pc*LMG2410 under iron-limiting conditions (12, 13), it is not cleaved at an appreciable rate by FusC and so accumulates intracellularly in wild-type *Pc*LMG2410 (Fig. 1b). To compare the levels of uptake of Fer_Pot_ in wild-type and Δ*fusC Pc*LMG2410, cells were grown under iron-limiting conditions through the addition of the iron chelator 2,2′-bipyridine and were supplemented with Fer_Pot_. The amount of Fer_Pot_ in whole-cell extracts and in the media was determined by immunoblotting. Levels of Fer_Pot_ obtained from cell extracts increased at the same rate in whole-cell extracts, and rates of removal of ferredoxin from the media were similar (Fig. 1c). These data show that FusC does not play a role in protein import and that the role of FusC in iron acquisition is likely restricted to proteolytic processing of ferredoxin as we previously reported (11).

To further probe the mechanism of ferredoxin uptake, we tested the ability of *Pc*LMG2410 cells to accumulate Fer_Pot_ under iron-limiting conditions and in the presence of the uncoupling agent carbonyl cyanide m-chlorophenylhydrazone (CCCP), which dissipates the PMF through transport of protons across the cytoplasmic membrane (18). The intracellular accumulation of Fer_Pot_ by *Pc*LMG2410 was markedly reduced in the presence of 10 μM CCCP, relative to cells grown in the absence of the uncoupling agent, and was abolished in the presence of 100 μM CCCP (Fig. 1d). Similar effects resulting from the action of CCCP were observed on the intracellular accumulation of *Arabidopsis* ferredoxin by Δ*fusC* LMG2410 (Fig. 1d). These data show that, analogously to other TBDT-mediated transport processes (10, 19), the import of ferredoxin is PMF dependent and a Brownian ratchet mechanism is unlikely to play a key role in ferredoxin import in *Pectobacterium* spp.

### FusB mediates ferredoxin import into the periplasm.

**FIG 2:**
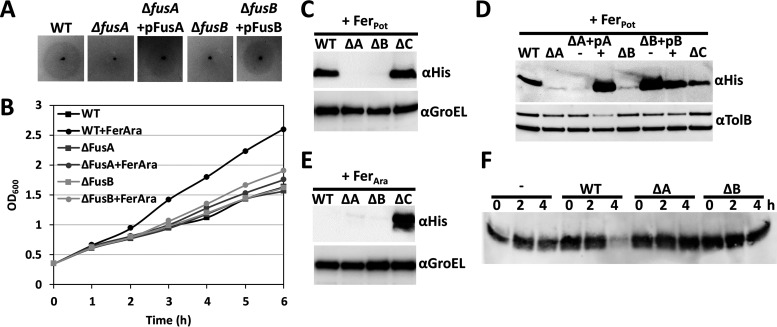
FusA and FusB are required for ferredoxin uptake. (A) Growth enhancement assay using spinach ferredoxin spotted onto a soft-agar overlay containing the wild-type (WT) strain, deletion strains, and deletion strains recomplemented in *trans* with the respective plasmids. Similar phenotypes were observed in 5 independent assays. (B) Growth curve comparing the rates of growth of the wild-type strain and each deletion strain in the absence and presence of *Arabidopsis* ferredoxin. Comparable rates were observed in 3 independent experiments. (C) Internalization assay of Fer_Pot_. Mid-log-phase cells of each strain were supplemented with 2,2′-bipyridine and 1 μM ferredoxin and grown for 1 h, and whole-cell extracts were probed with anti-His antiserum for His-tagged Fer_Pot_. GroEL served as the loading control. (D) Internalization assay of His-tagged Fer_Pot_, including deletion strains recomplemented in *trans* using plasmids harboring the corresponding genes. ΔA+pA, Δ*fusA* plus pFusA; ΔB+pB, Δ*fusB* plus pFusB. Prior to the addition of 2,2′-bipyridine and ferredoxin, the cultures of recomplemented strains were split in two and either supplemented with 0.5 mM IPTG (“+”) or grown in the absence of inducer (“-”). TolB served as the loading control. (E) Internalization assay using His-tagged Fer_Ara_ (see panel C). (F) Depletion assay showing the gradual reduction in the level of *Arabidopsis* ferredoxin in the media derived from uninoculated (-), wild-type, Δ*fusA* (ΔA), and Δ*fusB* (ΔB) cell cultures.

As we previously reported, in addition to *fusA* and *fusC*, the Fus operon carries genes that encode a TonB homologue, FusB, and an ABC transporter, FusD (12). Given the documented role of TonB in siderophore import in many bacterial species, we supposed that FusB might play a similar role in protein import, having perhaps evolved additional functionality required to mediate the passage of a large substrate through the lumen of the TBDT FusA. To test this hypothesis, we created Δ*fusA* and Δ*fusB* strains in *Pc*LMG2410 and initially probed them using growth enhancement assays under iron-limiting conditions. As indicated by the loss of growth enhancement both on solid media (Fig. 2a) and in liquid culture (Fig. 2b), these two genes encode proteins which are essential for Fus-mediated iron acquisition. The possibility that deletion of either gene affected the expression or level of FusC and thus indirectly affected the growth enhancement phenotype was ruled out by immunoblotting whole-cell extracts with anti-FusC antiserum (see Fig. S1 in the supplemental material). We further investigated the ability of the Δ*fusA* and Δ*fusB Pc*LMG2410 mutant strains to import ferredoxin relative to wild-type and Δ*fusC* strains using Fer_Pot_, which cannot be cleaved by FusC. Consistent with the hypothesized role of FusB in protein import, and in contrast to the wild-type and Δ*fusC* strains, we did not observe intracellular accumulation of Fer_Pot_ in Δ*fusA* and Δ*fusB Pc*LMG2410 (Fig. 2c). The ferredoxin import phenotype lost in the Δ*fusA* and Δ*fusB* strains was restored by plasmid-based complementation of *fusA* and *fusB*, respectively (Fig. 2d). In these experiments, the production of FusA and FusB was inducible by the use of IPTG (isopropyl-β-d-thiogalactopyranoside) under the control of the T5 promoter, although in the case of FusB complementation, leaky expression in the absence of IPTG is sufficient to restore protein import.

10.1128/mBio.02081-20.1 FIG S1 FusC is produced in Δ*fusA* and Δ*fusB* strains. The immunoblot shows the level of FusC (101 kDa) in the periplasmic fractions extracted from wild-type, Δ*fusA* (ΔA), Δ*fusB* (ΔB), and Δ*fusC* (ΔC) cells grown to mid-log phase in the presence of 2,2′-bipyridine. Download FIG S1, JPG file, 0.5 MB. Copyright © 2020 Wojnowska and Walker. 2020 Wojnowska and Walker. This content is distributed under the terms of the Creative Commons Attribution 4.0 International license.

To ensure that the abrogation of substrate import is not specific to Fer_Pot_, we also monitored the ability of the Δ*fusA* and Δ*fusB* strains to utilize Fer_Ara_. However, for this substrate, instead of measuring intracellular ferredoxin accumulation, we determined loss of ferredoxin from the growth media, since accumulation of Fer_Ara_ was observed only in the LMG2410 Δ*fusC* strain (Fig. 2e). Consistent with the internalization assay and growth enhancement assays, the ferredoxin content of the media decreased over time in the presence of wild-type cells but not in the presence of the Δ*fusA* and Δ*fusB* strains, indicating that both FusA and FusB are required for Fer_Ara_ uptake (Fig. 2f). To exclude the possibility that the increased depletion of ferredoxin from the media in the wild-type cultures was due to the higher growth rate of this strain than of the Δ*fusA* and Δ*fusB* strains in the presence of Fer_Ara_, we directly compared levels of Fer_Ara_ depletion from the media using the Δ*fusA* and Δ*fusC* strains (Fig. S2), as neither of these strains shows a ferredoxin-dependent growth enhancement. Over 7 h, Fer_Ara_ was gradually depleted from the media by the Δ*fusC* strain and accumulated in the cells, as indicated by immunoblots of whole-cell extracts obtained at the end of the assay. In contrast, the level of Fer_Ara_ in the media of the Δ*fusA* strain remained unchanged and no ferredoxin was detected in whole-cell extracts, providing further evidence that deletion of *fusA* (and *fusB*) abrogates the import of Fer_Ara._

10.1128/mBio.02081-20.2 FIG S2 Comparison of levels of Fer_Ara_ uptake by Δ*fusA* and Δ*fusC* cells. Upon reaching an OD_600_ of 0.5, both cultures were supplemented with 2,2′-bipyridine and Fer_Ara_ (1 μM) and grown for a further 7 h. Immunoblots show the level of ferredoxin present in the media at each time point and in the final whole-cell extracts at 7 h. GroEL served as the loading control for the whole-cell extracts. Download FIG S2, JPG file, 0.4 MB. Copyright © 2020 Wojnowska and Walker. 2020 Wojnowska and Walker. This content is distributed under the terms of the Creative Commons Attribution 4.0 International license.

### FusB directly interacts with the TonB box of FusA.

Having determined that the TonB-like protein FusB is required for ferredoxin import, we aimed to elucidate the mechanism of ferredoxin uptake. Analysis of the FusA sequence showed the presence of a putative TonB box, DTILVRST, with a sequence similar to that of TonB boxes from well-characterized Escherichia coli TBDTs and other *Pc*LMG2410 TBDTs (Fig. S3). The functional importance of this putative TonB box region was demonstrated using ferredoxin import assay and plasmid-based complementation of the Δ*fusA* strain, which showed that proline substitutions within the putative TonB box abolished internalization of the FusA substrate ferredoxin (Fig. S4). The similarity of the TonB box of FusA to the TonB boxes of other *Pc*LMG2410 and E. coli TBDTs suggests that FusA may interact with *Pc*LMG2410 TonB and not with FusB. Although *Pc*LMG2410 has 6 genes that encode TonB-like proteins, we hypothesized that the protein that was most similar to E. coli TonB, and that we refer to here as *Pc*TonB, would fulfil the same function as this protein in servicing multiple TBDTs, with the primary function of dislocating the plug domain to enable substrate transport.

10.1128/mBio.02081-20.3 FIG S3 Sequence alignment of the putative TonB box regions of FusA against other P. carotovorum (“Pc”) LMG2410 and E. coli TonB box regions. The numbers refer to the position of the predicted TonB box in the mature protein. Three P. carotovorum LMG2410 proteins were not included as their TonB boxes were not clearly identifiable. “Sm” refers to Serratia marcescens and “Ec” to E. coli. The consensus sequence shown at the bottom was generated using Weblogo. Download FIG S3, JPG file, 0.8 MB. Copyright © 2020 Wojnowska and Walker. 2020 Wojnowska and Walker. This content is distributed under the terms of the Creative Commons Attribution 4.0 International license.

10.1128/mBio.02081-20.4 FIG S4 Mutations within the putative FusA TonB box affect ferredoxin transport. (A) Potato ferredoxin import assay of Δ*fusA* cells recomplemented with a gene encoding either wild-type or FusA variants. (B) Outer membrane extracts confirming the expression and appropriate localization of wild-type and mutant FusA. Download FIG S4, JPG file, 0.6 MB. Copyright © 2020 Wojnowska and Walker. 2020 Wojnowska and Walker. This content is distributed under the terms of the Creative Commons Attribution 4.0 International license.

**FIG 3:**
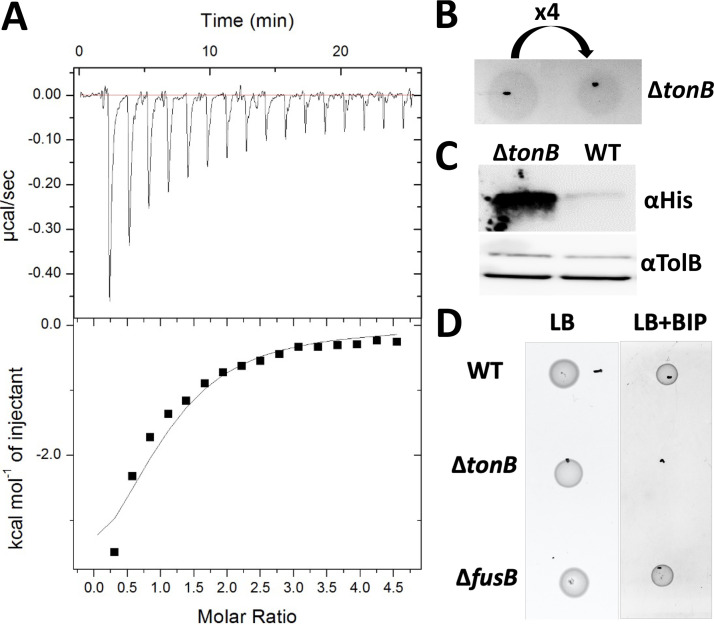
FusB interacts with the FusA “TonB box” and, unlike *Pc*TonB, is required for ferredoxin uptake. (A) ITC binding isotherm of 1 mM FusA_NTR_-GFP titrated into 90 μM FusB_CTD_. The calculated *K_d_* for the FusA_NTR_-GFP-FusB_CTD_ complex is 57 (±12) μM (*n* = 3). (B) Growth enhancement assay using spinach ferredoxin on soft-agar overlay containing Δ*tonB* cells. “x4” refers to the dilution factor. The experiment was repeated 5 times with comparable outcomes. (C) Uptake of His-tagged potato ferredoxin by Δ*tonB* and wild-type cells. Whole-cell extracts obtained after 1 h by incubation of each cell type with ferredoxin were probed with anti-His antiserum. TolB served as the loading control. The experiment was performed 4 times with comparable results. (D) Growth assay of wild-type (WT), Δ*tonB*, and Δ*fusB* cells spotted onto LB agar (LB) or LB agar supplemented with 400 μM 2,2′-bipyridine (LB+BIP). Comparable phenotypes were observed in two independent experiments.

To determine if TonB or FusB or both TonB and FusB interact directly with FusA, we produced a construct consisting of the N-terminal region of FusA (residues 21 to 66), excluding the signal peptide region, fused to green fluorescent protein (GFP) (FusA_NTR_-GFP) and determined if this interacts with the isolated C-terminal domains of *Pc*TonB (TonB_CTD_) and FusB (FusB_CTD_) by isothermal titration calorimetry (ITC). Clearly identifiable heat data representing binding were observed on titration of FusA_NTR_-GFP into FusB_CTD_, although the affinity of FusB_CTD_ for FusA_NTR_-GFP is weak (57 μM) (Fig. 3a). No heat data representing binding were observed on titration of isolated GFP into FusB_CTD_ (Fig. S5), showing that the C-terminal domain of FusB specifically interacts with the N-terminal region of FusA. Interestingly, similar heat data representing binding were observed on titration of FusA_NTR_-GFP into TonB_CTD_ (Fig. S6), demonstrating that the N-terminal region of FusA can also interact with *Pc*TonB.

10.1128/mBio.02081-20.5 FIG S5 GFP does not interact with FusB_CTD_. Data represent titration of 1.2 mM GFP, resulting from TEV-mediated cleavage of FusA_NTR_-GFP, into 60 μM FusB_CTD_. Download FIG S5, JPG file, 0.4 MB. Copyright © 2020 Wojnowska and Walker. 2020 Wojnowska and Walker. This content is distributed under the terms of the Creative Commons Attribution 4.0 International license.

10.1128/mBio.02081-20.6 FIG S6 TonB_CTD_ forms a complex with the N-terminal domain of FusA. ITC was performed by titrating 1.2 mM FusA_NTR_-GFP into 45 μM TonB_CTD_. Download FIG S6, JPG file, 0.2 MB. Copyright © 2020 Wojnowska and Walker. 2020 Wojnowska and Walker. This content is distributed under the terms of the Creative Commons Attribution 4.0 International license.

To determine if the *Pc*TonB plays a role in ferredoxin uptake, we deleted the corresponding gene in *Pc*LMG2410 and tested the growth enhancement phenotype of this strain in the presence of Fer_Sp_. In contrast to deletion of *fusB*, the loss of *tonB* did not reduce the growth enhancement phenotype (Fig. 3b); in fact, the *ΔtonB* strain showed zones of growth enhancement in the presence of Fer_Sp_ that were more prominent than those seen with the wild-type strain and showed increased intracellular accumulation of Fer_Pot_ relative to wild-type *Pc*LMG2410 (Fig. 3c). However, *Pc*LMG2410 *ΔtonB* exhibited poor growth in the presence of 2,2′-bipyridine relative to the wild-type strain (Fig. 3d), with very faint growth observable after 24 h. These data indicate that, although *Pc*TonB does play the expected generic role in iron uptake, this does not include iron acquisition from ferredoxin. Furthermore, despite the aforementioned observation that TonB interacts with FusA *in vitro* (and possibly *in vivo*), this interaction is not sufficient for ferredoxin uptake. Indeed, there may be competition between FusB and TonB for complex formation with FusA, with only the FusB-FusA complex being productive with respect to ferredoxin uptake.

### FusB interacts directly with the ferredoxin substrate.

**FIG 4:**
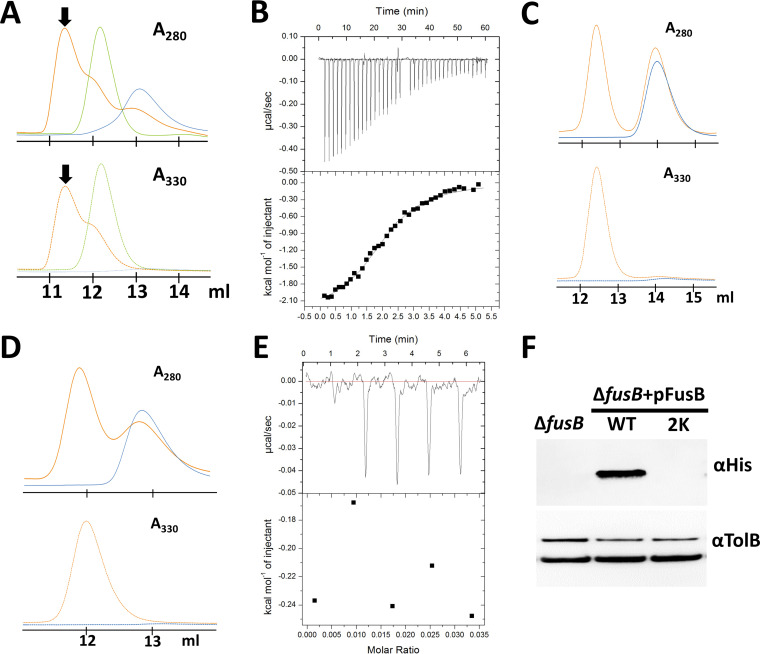
FusB interacts with ferredoxin substrate. (A) Overlaid size exclusion chromatograms of FusB_CTD_ (blue) and spinach ferredoxin (green) and a mixture of the two proteins (orange), with the arrows pointing at the ferredoxin-FusB_CTD_ complex. Similar results were obtained from 5 independent runs. (B) ITC binding isotherm of 600 μM Fer_Sp_ titrated into 70 μM FusB_CTD_. The calculated *K_d_* value for the Fer_Sp_-FusB_CTD_ complex is 8.7 (±3.5) μM (*n* = 3). (C) TonB_CTD_ does not form a complex with ferredoxin. Overlay of size exclusion chromatograms shows TonB_CTD_ in the absence (blue trace) and in the presence (orange trace) of Fer_Sp_. (D) Size exclusion chromatograms (280 and 330 nm) of FusB_CTD_ R176K/R177K alone (blue) or in the presence of spinach ferredoxin (orange) showing no complex formation. (E) ITC titration of Fer_Sp_ into FusB_CTD_ R176K/R177K showing only heat data correlating to dilution. (F) Ferredoxin import assay showing the level of uptake of His-tagged potato ferredoxin by Δ*fusB* cells complemented with plasmids encoding either wild-type (WT) or FusB_CTD_ R176K/R177K (2K). The experiment was performed 2 times.

The ability of FusB and *Pc*TonB to interact with FusA, but with only the former able to mediate ferredoxin uptake, suggests that FusB plays an additional role essential for ferredoxin import. One possibility is that FusB directly interacts with the protein substrate after the initial binding of ferredoxin to FusA at the cell surface. To test this, we sought to determine if ferredoxin forms a complex with the isolated C-terminal domain of FusB by size exclusion chromatography (SEC). SEC of ferredoxin mixed with FusB_CTD_ monitored at 280 nm and 330 nm gave a peak that indicated the presence of a species of higher molecular weight than FusB_CTD_ or Fer_Sp_ alone, providing evidence of complex formation (Fig. 4a). We further investigated the formation of the FusB_CTD_-Fer_Sp_ complex by isothermal titration calorimetry, titrating Fer_SP_ into FusB_CTD_ (Fig. 4b). These data show that FusB interacts directly with the ferredoxin substrate. In contrast, no complex formation was observed between ferredoxin and the purified C-terminal domain of TonB (TonB_CTD_) using SEC (Fig. 4c).

Inspection of the amino acid sequence of FusB shows that the N-terminal portion of the predicted globular domain and the preceding linker contained a significant number of positively charged amino acids; such amino acids were absent from *Pc*LMG2410 and E. coli proteins (Fig. S7). Two such residues (Arg176 and Arg177) were found in place of the highly conserved Gln-Pro-Gln residues, which form a part of the BtuB TonB box binding motif (QPQYP) in E. coli TonB (20). This arginine motif is located within a loop/linker region of TonB proteins, connecting the periplasm-spanning and globular domains. Substitution of the two FusB_CTD_ arginine residues with lysines rendered a folded protein that did not comigrate with ferredoxin in SEC (Fig. 4d), and no heat data representing binding were detected by ITC on titration of Fer_Sp_ into FusB_CTD_ R176K/R177K (Fig. 4e). Similarly, *Pc*LMG2410 Δ*fusB* could not be complemented with a pFusB plasmid encoding the FusB R176K/R177K variant (Fig. 4f). Therefore, at least one of these two arginine residues appears to be critical for FusB-substrate interaction.

10.1128/mBio.02081-20.7 FIG S7 ClustalW sequence alignment of the C-terminal domains of FusB, *Pc*TonB, and *Ec*TonB. Alignments started at residue 211 of FusB, residue 158 of *Pc*TonB, and residue 140 of *Ec*TonB. Black arrows indicate the highly conserved Tyr and Pro residues; red ones show arginines in FusB that were present in the first loop of the C-terminal domain. Download FIG S7, JPG file, 0.6 MB. Copyright © 2020 Wojnowska and Walker. 2020 Wojnowska and Walker. This content is distributed under the terms of the Creative Commons Attribution 4.0 International license.

## DISCUSSION

Our recent discovery that ferredoxin is imported into the periplasm of *P. carotovorum* revealed an unprecedented example of protein uptake for nutrient acquisition in Gram-negative bacteria (11). In this work, we define key aspects of the mechanism of ferredoxin transport across the outer membrane. In a recent report, it was hypothesized that the M16 protease FusC acts as a periplasmic anchor that facilitates ferredoxin uptake by means of Brownian-ratchet mechanism (15). However, the data presented here are inconsistent with this model, showing that ferredoxin import is independent of FusC. Instead, ferredoxin uptake requires energy transduction from the PMF and the TonB-like protein FusB. Therefore, the mechanism of ferredoxin import shares some similarity with the mechanism of import of widely studied substrates of TBDTs, such iron siderophores and vitamin B12 (4, 21). For these substrates, according to the currently accepted models of TonB-dependent transport, the major role of TonB is in the displacement or partial displacement of the plug domain from their specific TBDTs (6, 10).

Interestingly, in the case of FusA, both FusB and *Pc*TonB are able to interact with its N-terminal region and so both these proteins may be able to facilitate displacement of the FusA plug domain. However, deletion of the genes encoding the two TonB proteins showed that only FusB is essential for ferredoxin transport, demonstrating an additional role for FusB in this process that cannot be fulfilled by *Pc*TonB. Although the affinity of FusB_CTD_ for FusA_NTR_ is low (57 μM), comparable complexes showing low affinity between TBDT TonB-binding peptides and TonB proteins have been previously described. For example, the affinity reported for the TonB-like protein HasB interaction with a 21-mer HasR N-terminal peptide was 25 μM (22). Similarly weak interactions between TonB and TBDT TonB box peptides have been reported for FhuA (36 μM) (23) and BtuB (9.4 μM) (6). However, complex formation between TonB and TonB-binding peptides is characterized by β-strand augmentation, which is known to result in the formation of mechanically strong complexes (6). Indeed, it has been demonstrated *in vitro* using atomic force microscopy that the TonB-BtuB Ton box complex is sufficiently mechanically robust to induce partial unfolding of the BtuB plug domain, forming a channel through which the vitamin B12 substrate can translocate (6).

**FIG 5:**
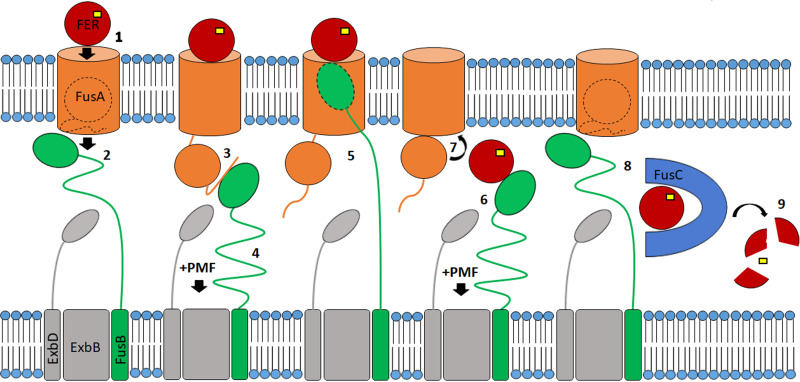
Proposed mechanism of FUS-mediated ferredoxin import mechanism. In the proposed mechanism, FusB (green) fulfils two roles, first, in displacement of the FusA plug domain, and, second, in directly mediating ferredoxin translocation via the FusA lumen. Binding of ferredoxin (red) to FusA at the cell surface (step 1) causes release of the FusA TonB box into the periplasm (step 2), where it is bound by FusB, which dislocates the plug domain (step 3) through energy transduced from the PMF via the ExbBD complex (step 4). FusB is then able to enter the lumen of FusA and bind ferredoxin (step 5) and, through transduction of the PMF, translocate ferredoxin into the periplasm (step 6). The plug domain is then able to reenter the FusA barrel (step 7) and return the FusA and FusB proteins to their resting states. Ferredoxin is then bound by FusC in the periplasm (step 8), which proteolytically cleaves the substrate, releasing the iron-sulfur cluster (yellow) (step 9).

The ability of FusB to form a complex with ferredoxin, which *Pc*TonB lacks, indicates that this additional role involves the direct interaction of FusB with the ferredoxin substrate and that this complex formation is essential for ferredoxin transport through the lumen of FusA. Consistent with this, we identified an arginine motif that is required for FusB-mediated ferredoxin uptake by *P. carotovorum* and formation of the FusB-ferredoxin complex. Our current model of Fus-mediated iron acquisition, whereby FusB fulfils two distinct roles, is schematically shown in Fig. 5. In this model, binding of the substrate on the extracellular side of FusA releases the TonB box into the periplasmic space, where it is captured by FusB. Due to the dimensions of the globular ferredoxin, which are similar to those of the lumen of its FusA TBDT (11), ferredoxin is unlikely to be able to readily diffuse into the periplasm after removal of the FusA plug domain. We therefore hypothesize that the interaction of FusB with the substrate involves a further PMF-dependent step required to pull the ferredoxin substrate through the lumen of FusA. This would involve the C-terminal domain of FusB, which is of a size comparable to those of plant ferredoxins, entering the lumen of FusA to contact ferredoxin on the cell surface. The FusB-ferredoxin complex would then be able to be pulled into the periplasm, using the ExbBD complex and PMF, after which the substrate would be processed by FusC. Although we present a model relying on a single FusB protein per import cycle, we cannot exclude the possibility that the removal of the FusA plug and ferredoxin import would involve two separate FusB molecules.

The occurrence of genes encoding multiple TonB-like proteins is a common feature of many Gram-negative bacteria (24), and specific TonB proteins are required in some cases for the uptake of specific substrates, as in the case of TonB2 of Vibrio anguillarum for anguibactin uptake (25), while other cases exhibit some level of functional redundancy (26). However, to our knowledge the Fus system represents the only substrate import system in which a TonB protein has been shown to directly interact with the substrate. This additional functionality displayed by FusB may reflect the nature of the ferredoxin substrate, which is atypically large in comparison to the well-studied TBDT siderophore substrates. In this respect, the uptake of ferredoxin is similar to the TonB-dependent uptake of the colicins and pyocins, which directly interact with TonB after threading their TonB box-containing intrinsically unstructured translocation domain (IUTD) though the lumen of the corresponding TBDT (10, 27). However, since plant ferredoxins are highly stable proteins that lack any kind of similar unstructured regions, our hypothesis is that in order to contact ferredoxin, FusB must enter the FusA lumen and contact FusA-bound substrate at the cell surface. This proposed mechanism also explains why the ferredoxin-containing bacteriocins do not require an IUTD that contains a TonB box to cross the *P. carotovorum* outer membrane, with FusB proteins able to directly contact their ferredoxin receptor-binding domains at the cell surface, thus enabling parasitization of the Fus system (12, 14).

In summary, we describe a novel mechanism of TonB-dependent nutrient uptake that requires a direct interaction between the substrate and the cognate TonB protein. The occurrence of multiple TonB proteins in many Gram-negative bacteria suggests that similar mechanisms may operate for atypical TBDT substrates.

## MATERIALS AND METHODS

### Bacterial strains and media.

E. coli was grown in LB broth or plated on LB agar and grown at 37°C. DH5α and BL21(DE3) strains were used as host strains for cloning and for IPTG-induced protein expression, respectively. *P. carotovorum* was grown in LB broth or plated on LB agar at 30°C with the addition of the iron chelator 2,2′-bipyridine where specified. LB media and agar for culturing plasmid-complemented deletion strains always contained 100 μg ml^−1^ ampicillin.

### Generation of gene knockout strains and plasmids.

The *fusA* (KAA3668913), *fusB* (KAA3668912), *fusC* (KAA3668914), and *tonB* (KAA3668374) sequences were determined from the genome sequence of *P. carotovorum* LMG2410 (GenBank BioProject accession no. PRJNA543207) (28). Genes of *Pc*LMG2410 were deleted using the lambda red method as described previously (11, 29). The primers used for amplifying the kanamycin cassette from pKD4 template plasmid and gene sequences from genomic DNA and for plasmid site-directed mutagenesis are listed in Table S1 in the supplemental material. The gene knockouts were confirmed by PCR and sequencing. Table S2 shows all the plasmids used in this study. To construct all of the plasmids, except pFusANTR-GFP, the respective genes were amplified from wild-type genomic DNA using primers that contained flanking regions with NdeI (forward) and XhoI (reverse) restriction enzyme sites. Purified PCR products were digested and ligated into NdeI/XhoI-digested pJ404, which carries ampicillin resistance. To generate pFusANTR-GFP, the sequence encoding the N-terminal portion of FusA was amplified with primers containing XhoI (forward) and BamHI (reverse) restriction enzyme sites and the PCR products were inserted into XhoI/BamHI-digested pWaldo plasmid (30). The complementation plasmids were transformed into competent LMG2410 knockout strains by electroporation.

10.1128/mBio.02081-20.8 TABLE S1 List of primers used in this study. Download Table S1, DOCX file, 0.01 MB. Copyright © 2020 Wojnowska and Walker. 2020 Wojnowska and Walker. This content is distributed under the terms of the Creative Commons Attribution 4.0 International license.

10.1128/mBio.02081-20.9 TABLE S2 List of plasmids used in this study. Download Table S2, DOCX file, 0.01 MB. Copyright © 2020 Wojnowska and Walker. 2020 Wojnowska and Walker. This content is distributed under the terms of the Creative Commons Attribution 4.0 International license.

### Protein production and purification.

FusC, FusB_CTD_, TonB_CTD_, FusA_NTR_-GFP, and all ferredoxin proteins were overproduced in E. coli and purified as described previously (11, 12), with the exception of spinach ferredoxin (Fer_Sp_), which was purchased from Sigma. GFP alone used as a negative control in ITC was produced by cleavage of FusA_NTR_-GFP with tobacco etch virus (TEV) protease for 2 h at room temperature (RT) at a 50:1 ratio. The resulting GFP-His_8_ was separated from residual TEV protease by size exclusion chromatography, and the removal of the N-terminal region of FusA was confirmed by SDS-PAGE.

### Growth enhancement assays.

Growth enhancement in the presence of ferredoxin was performed on solid media as previously described (11). Briefly, 10 ml of 0.8% precooled agar was supplemented with 50 μl of mid-log culture in LB media and poured onto an LB agar base containing 400 μM 2,2′-bipyridine (and 0.2 mM IPTG where specified). For plasmid based complementation, Ampicillin (100 μg/ml) was added to the LB media base. A 4-μl volume of ferredoxin was spotted onto the solidified plate at the specified concentration. For growth enhancement in liquid media, bacteria were grown in M9 minimal media. Cultures (10 ml) were inoculated with a 1-in-50 dilution of overnight LB cultures and, upon reaching an optical density at 600 nm (OD_600_) of 0.45, were supplemented with 0.2 μM Fer_Ara_, and growth was monitored by measuring the OD_600_ for 6 h.

### Ferredoxin internalization and depletion assays.

All experiments were repeated at least once. The time course of ferredoxin internalization was initiated by supplementing LB cultures of wild-type or Δ*fusC* cells (OD_600_ of 0.5) with 2,2′-bipyridine to reach a final concentration of 200 μM. Fer_Ara_ or Fer_Pot_ was added to reach a final concentration of 1 μM, and the cultures were grown at 30°C with shaking over the specified time. At each time point, a volume equivalent to 1 ml of cell suspension at OD_600_ of 0.5 was removed and the cells were spun down and treated with BugBuster (Merck) for extraction of soluble protein. To determine if the Δ*fusA* and Δ*fusB* strains could take up ferredoxin, LB cultures of WT and deletion strains at an OD of ∼0.5 were supplemented with 200 μM 2,2′-bipyridine and 1 μM *Arabidopsis* or 5 μM potato ferredoxin. After 2 h at 30°C with shaking, 1 ml of cells was pelleted and soluble proteins were extracted using BugBuster (Merck). For internalization experiments involving plasmid-complemented deletion strains, LB cultures were grown until an OD_600_ of 0.4 was reached, whereupon 2,2′-bipyridine and potato ferredoxin were added. Δ*fusA*+pFusA and Δ*fusB*+pFusB cultures were split into two separate tubes, one of which was supplemented with IPTG to reach a final concentration of 0.2 mM. After 2 h, cells were harvested and subjected to BugBuster extraction as described above.

Depletion of *Arabidopsis* ferredoxin was monitored in 2 ml M9 minimal medium cultures of WT, Δ*fusA*, and Δ*fusB* strains over the course of 4 h. Each culture, as well as 2 ml of uninoculated media (negative control), was supplemented with 100 μM 2,2′-bipyridine and 1 μM Fer_Ara_. At each time point, 50 μl of culture was removed from each tube, and after the cells were pelleted, the supernatant was mixed with SDS loading dye.

The effect of dissipating PMF on ferredoxin uptake was determined using protonophore CCCP (Sigma), which was dissolved in dimethyl sulfoxide (DMSO) to reach a final concentration of 10 mM. Mid-log cultures of wild-type and Δ*fusC Pc*LMG2410 in M9 media were supplemented with 300 μM 2,2′-bipyridine, and 2 ml of each culture was mixed with 18 μl DMSO and 2 μl CCCP stock (for a 10 μM CCCP final concentration) or 20 μl CCCP stock (for a 10 μM final CCCP concentration) or with 20 μl DMSO alone for the “no-CCCP” control. The cultures were mixed and incubated at room temperature for 10 min, after which the wild-type cultures were supplemented with 1 μM Fer_Pot_ and the Δ*fusC* cultures with 0.2 μM Fer_Ara_. After 45 min of incubation at 30°C with shaking, 1 ml of each culture was pelleted, washed with 0.5 ml phosphate-buffered saline (PBS), and subjected to BugBuster extraction.

### Ferredoxin cleavage assays.

Cleavage reactions were performed at RT in a mixture containing 10 mM Tris-HCl (pH 7.5), 50 mM NaCl, 2 μM FusC, and 250 μM ferredoxin. At each time point, a 12-μl volume was removed and mixed with SDS loading dye. Proteins were resolved on a 16% SDS-PAGE gel and visualized by Coomassie staining.

### Analytical size exclusion chromatography.

Proteins were concentrated to ∼600 μM, and 20 μl of TonB_CTD_ or the relevant construct of FusB_CTD_ was mixed with an equal volume of Fer_Sp_. The mixtures were then diluted with SEC buffer (20 mM Tris-HCl [pH 7.5], 150 mM NaCl) to 0.2 ml and loaded onto Superdex 75 10/300 column (GE Healthcare), preequilibrated in the same buffer. Each protein was also passed through the column individually for reference. The chromatograms were recorded at both 280 and 330 nm.

### Isothermal titration calorimetry.

Experiments were performed on a MicroCal iTC_200_ instrument (Malvern) at 25°C in 10 mM Tris-HCl (pH 7.5)–150 mM NaCl, with a differential power setting of 3. All proteins were dialyzed against the ITC buffer overnight at 4°C with the exception of FusB_CTD_ and TonB_CTD_, which were subjected to gel filtration in ITC buffer immediately before the experiment. Each type of titration was repeated at least once using different batches of purified proteins. Injection volumes of 2 μl were used, and the titrations were continued until the signal corresponded to heat data representing dilution. The magnitude of heat data representing dilution for each titrant was established in a separate experiment, where the titrant was injected into buffer. Dissociation constant (*K_d_*) values are expressed as means (± standard errors of the means [SEM]).
