# Supplementary figures and images for: FusB Energizes Import across the Outer Membrane through Direct Interaction with Its Ferredoxin Substrate
Source: mBio. 2020 Oct 27;11(5):e02081-20. doi: 10.1128/mBio.02081-20 (PMC7593965; doi:10.1128/mBio.02081-20)

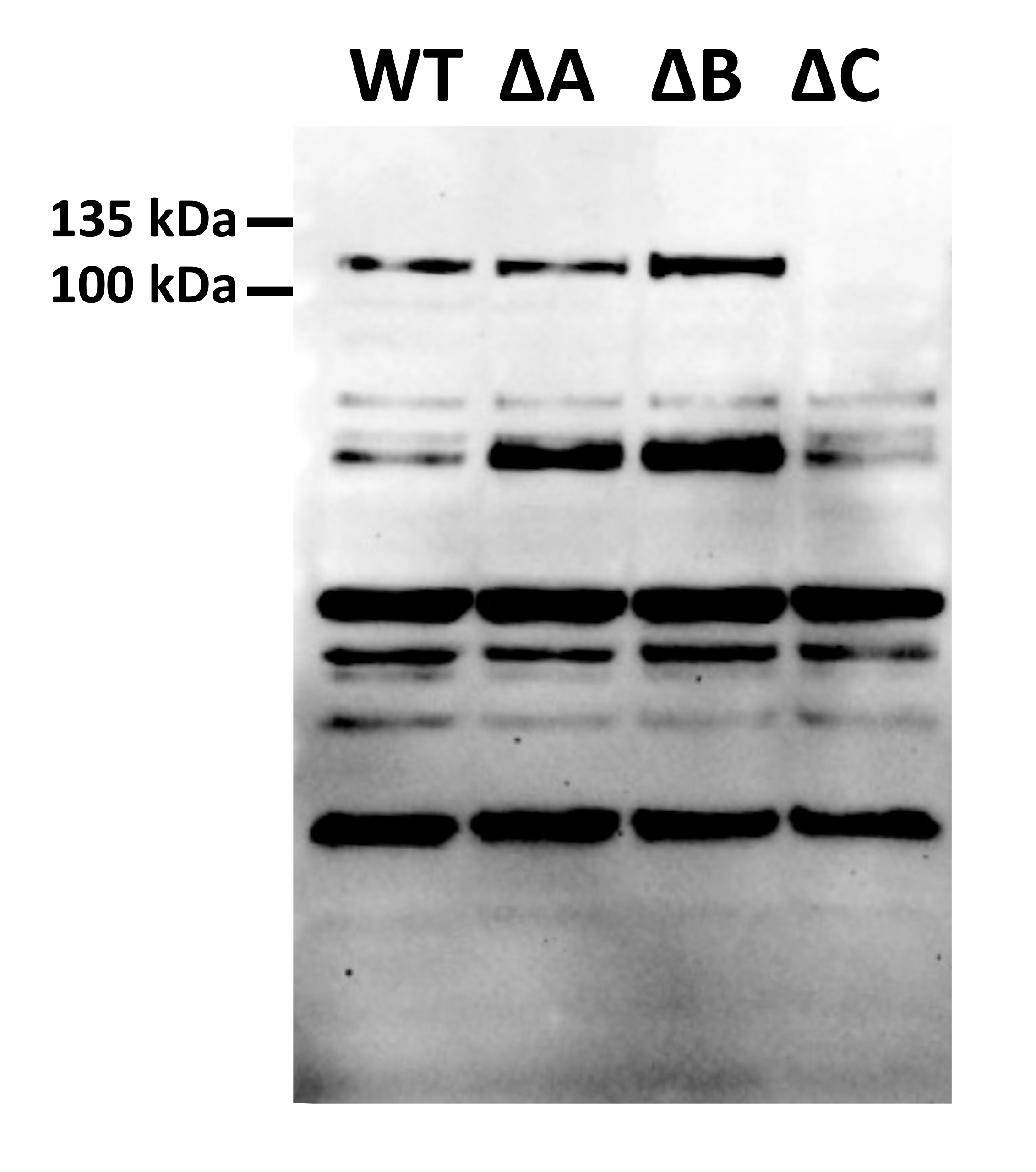

Supplement: FIG S1 [file mBio.02081-20-sf001.jpg]

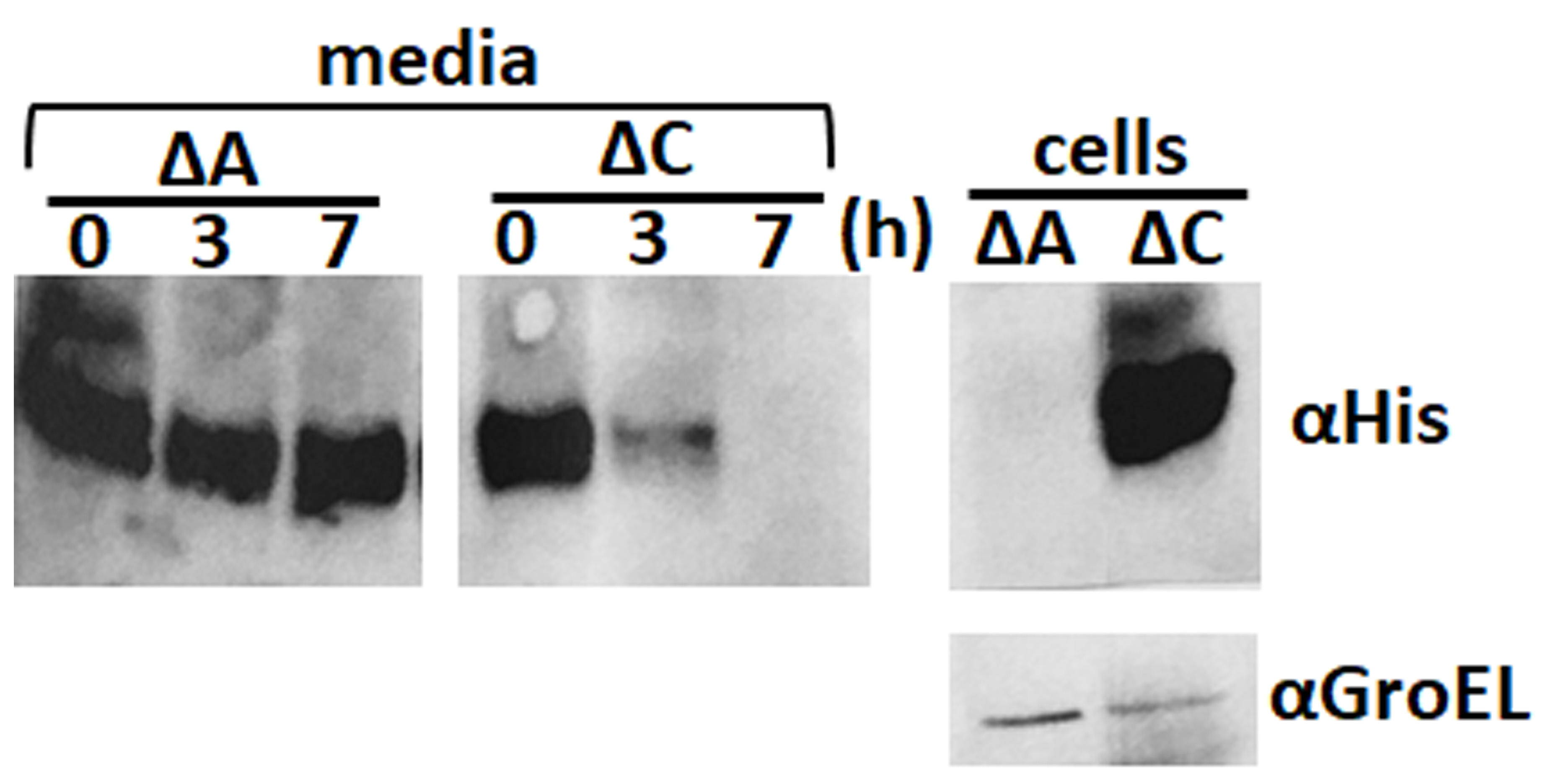

Supplement: FIG S2 [file mBio.02081-20-sf002.jpg]

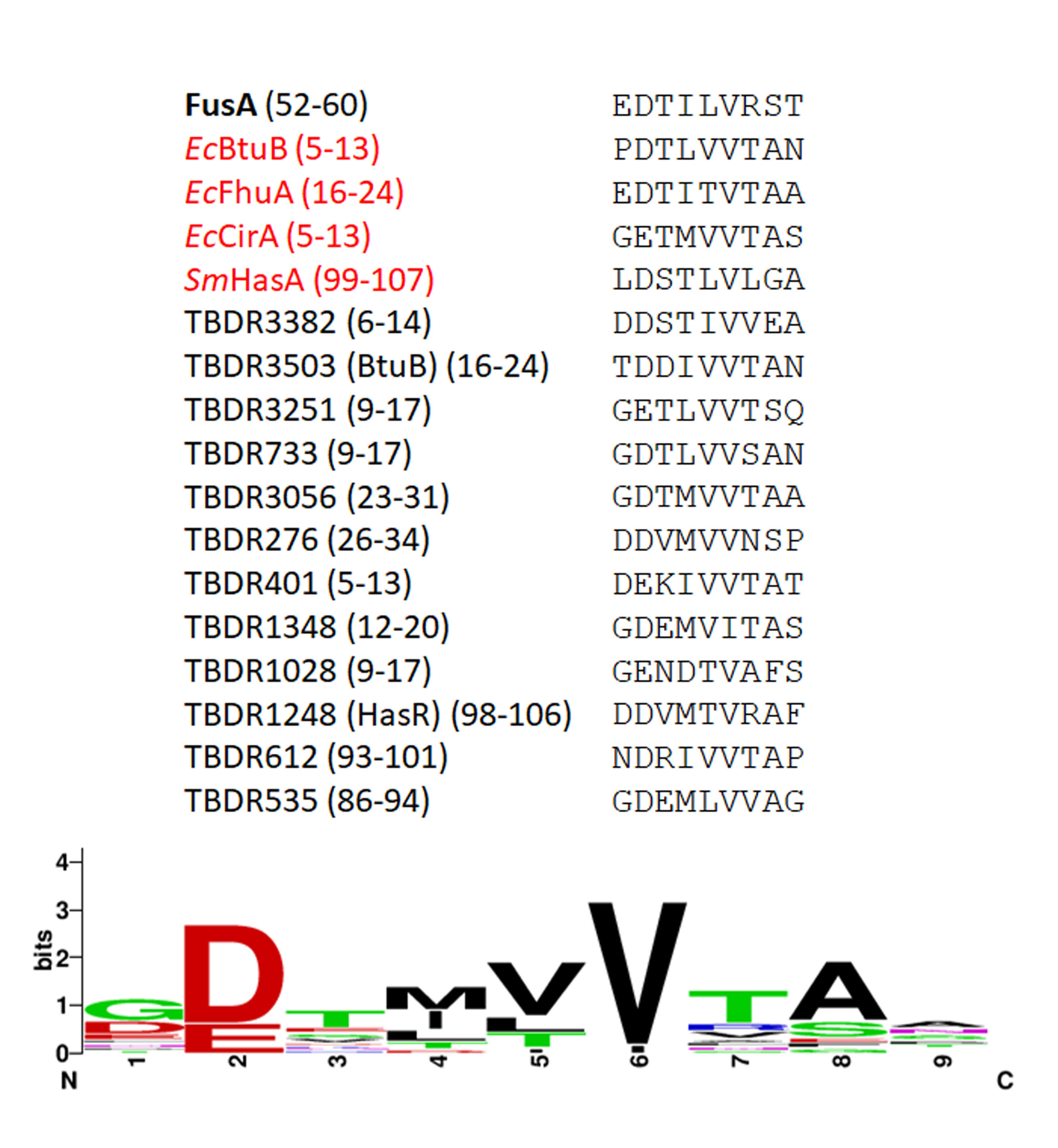

Supplement: FIG S3 [file mBio.02081-20-sf003.jpg]

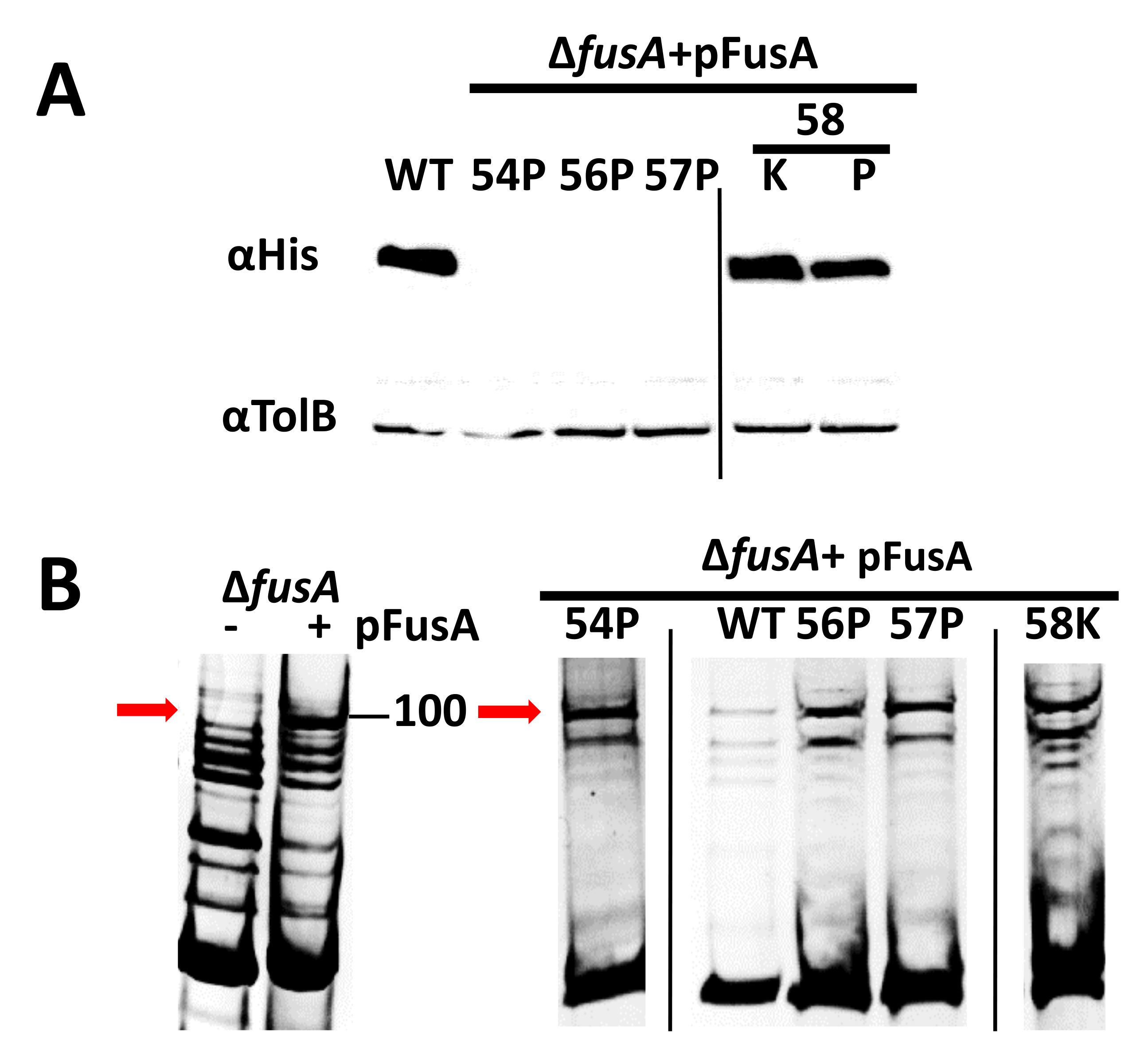

Supplement: FIG S4 [file mBio.02081-20-sf004.jpg]

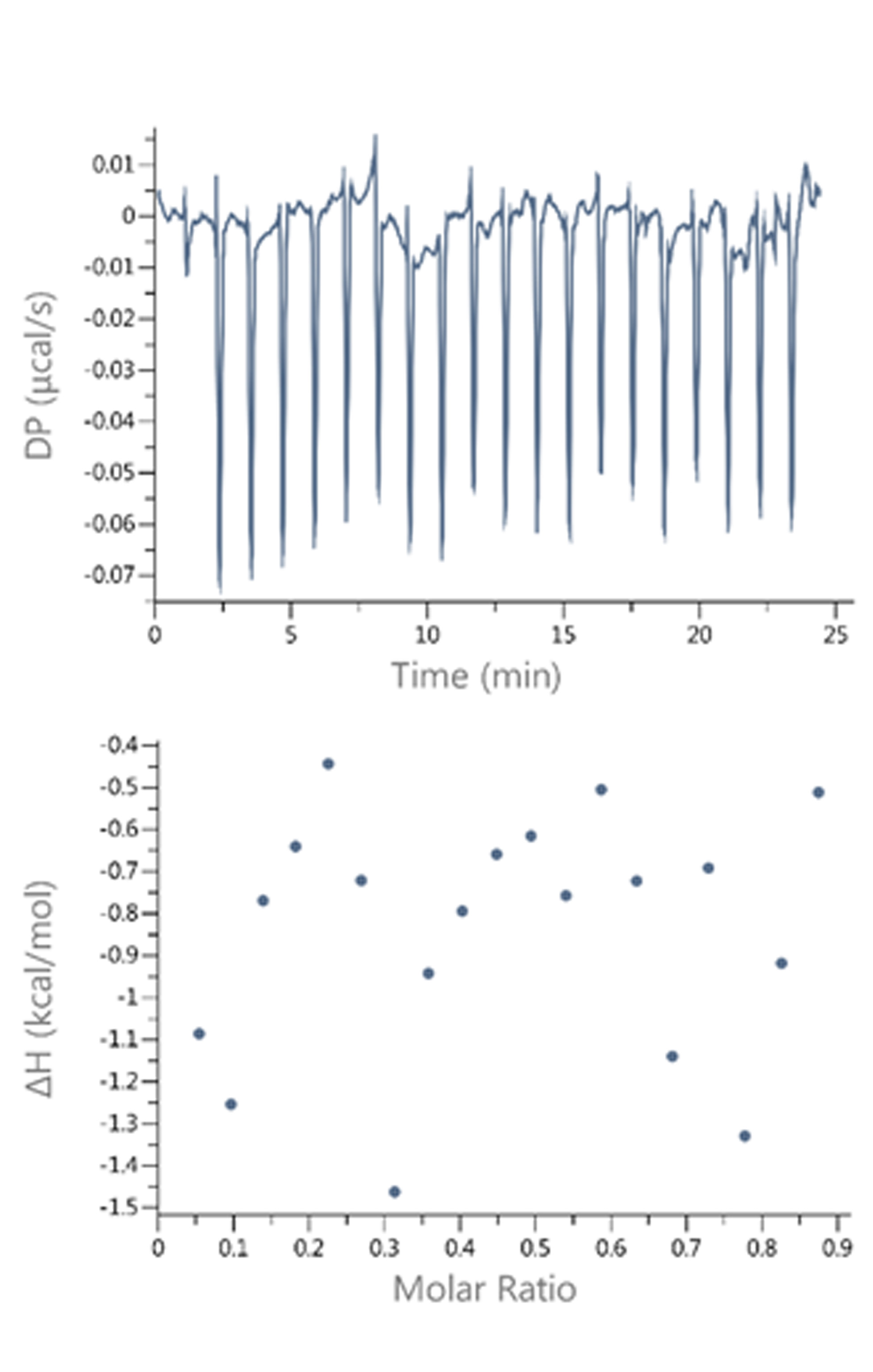

Supplement: FIG S5 [file mBio.02081-20-sf005.jpg]

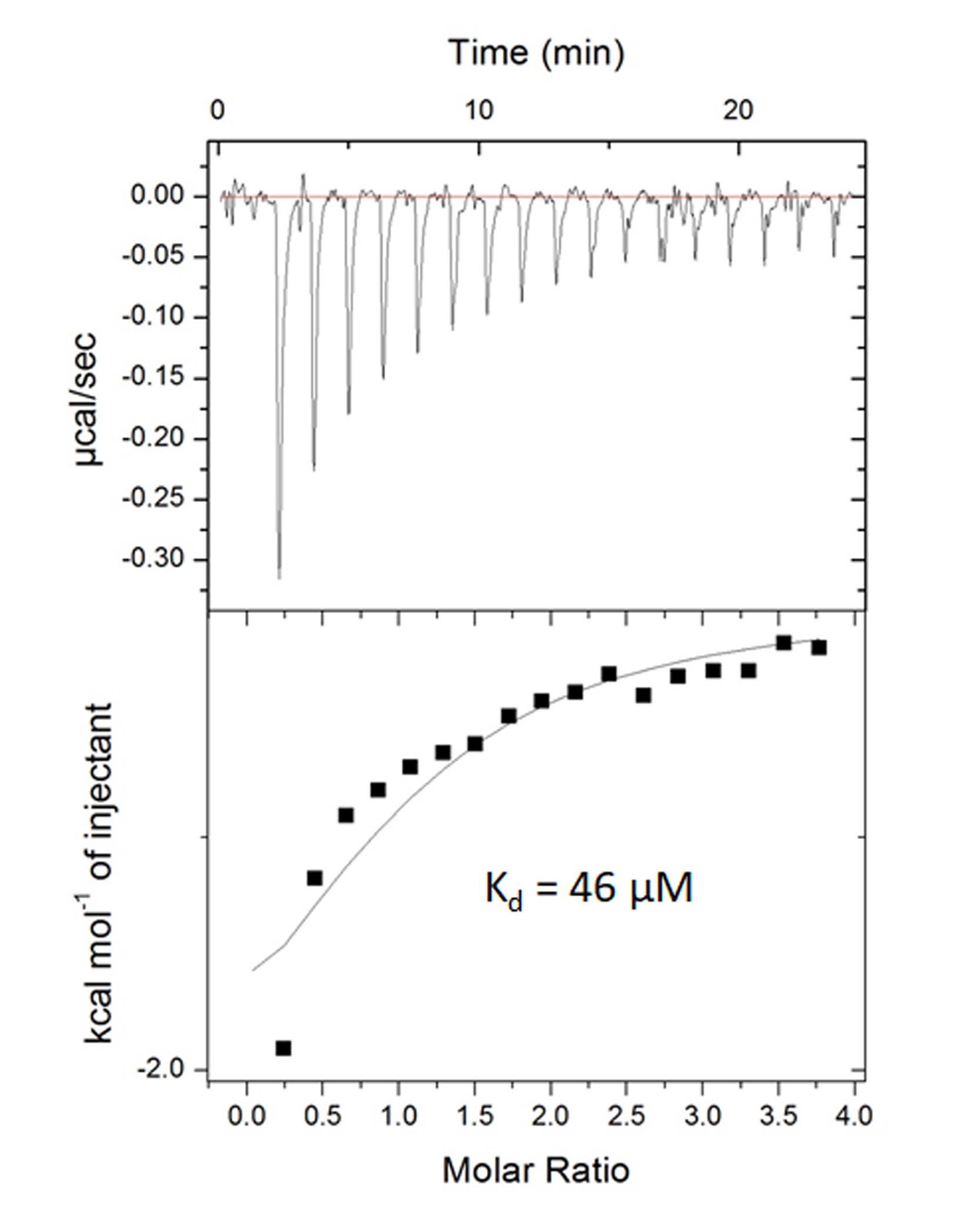

Supplement: FIG S6 [file mBio.02081-20-sf006.jpg]

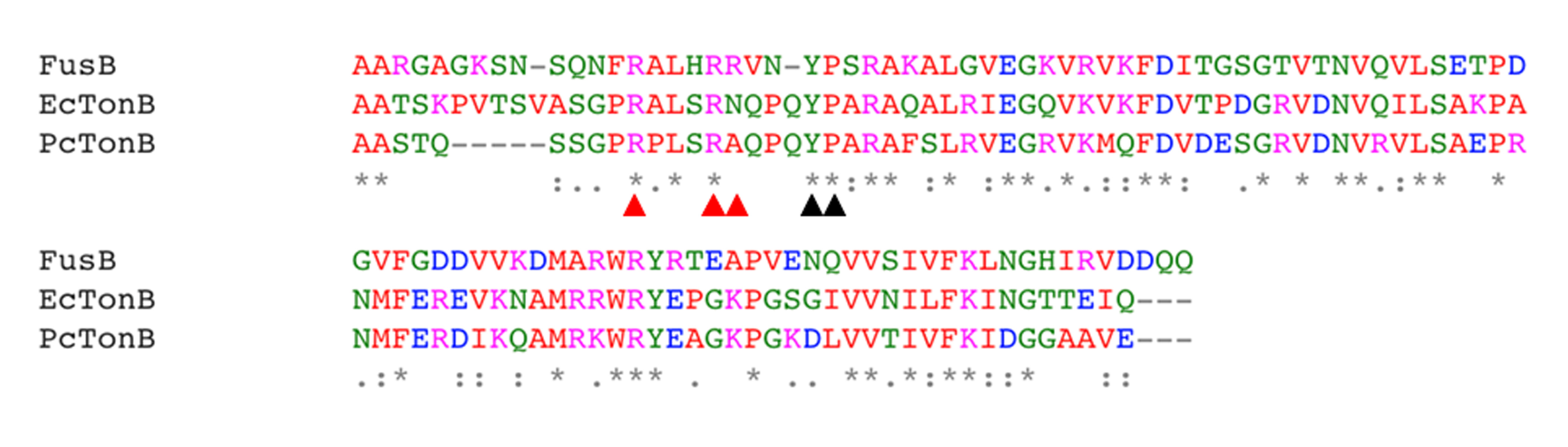

Supplement: FIG S7 [file mBio.02081-20-sf007.jpg]
