# Supplementary material for: FusB Energizes Import across the Outer Membrane through Direct Interaction with Its Ferredoxin Substrate
Source: mBio. 2020 Oct 27;11(5):e02081-20. doi: 10.1128/mBio.02081-20 (PMC7593965; doi:10.1128/mBio.02081-20)
Supplement: TABLE S1 [file mBio.02081-20-st001.docx]

**Table S1. List of primers used in this study.**

| **Target plasmid/**  **gene** | **Primer name** | **Primer sequence (5’🡪3’)** |
| --- | --- | --- |
| fusA knockout | FusAKOfor | cacatggaatttacaataatattttatttttaaaatgattatgcGTGTAGGCTGGAGCTGCTTC |
|  | FusAKOrev | ggaTttaccaggtgtaagcgacgccaagccagaactgacggccCATATGAATATCCTCCTTAG |
| fusB knockout | FusBKOfor | CtgcactactgacaccaggaagttgaagtttaagagatggagttggGTGTAGGCTGGAGCTGCTTC |
|  | FusBKOrev | cccaataagcataatcattttaaaaataaaatattattgtaaattccCATATGAATATCCTCCTTAG |
| TonB knockout | TonBKOfor | cgtgatgttgtttgaagacaatagtgcgaggacaataggttgGTGTAGGCTGGAGCTGCTTC |
|  | TonBKOrev | CGCGCTCGAACATATTGCGTGGCTCAGCAGAAAGCACGCGCACGCATATGAATATCCTCCTTAG |
| ExbB knockout | ExbBKOfor | gtacagaacagatgattgtacagaacgaatggtggaggcacagcGTGTAGGCTGGAGCTGCTTC |
|  | ExbBKOrev | cgttgctggcagccacatcgagatcgcgactttgcagcagcagCATATGAATATCCTCCTTAG |
| pFusA | FusAfor | GGAATTCcatAtgaataagaacgtctatttaatg |
|  | FusA rev | cagctcgagttaccaggtgtaagcgac |
| pFusB | FusBfor | GGAATTCcatatgagtagtgaaaatcttc |
|  | FusBrev | ctgctcgagttattgctggtcatcaac |
| pFusB-CTD | FusBCTDfor | GGAATTCcatatggcggcaacggccaagg |
|  | FusBCTDrev | cacctcgagttgctggtcatcaac |
| pTonB-CTD | TonBCTDfor | GGAATTCcatatggcagcaagcacgcagtc |
|  | TonBCTDrev | cacctcgagTTCGACTGCCGCCCCGCC |
| pExbB | ExbBfor | GGAATTCcatatgaagacggctgtcagtaatac |
|  | ExbBrev | cacctcgagttaacccacccgcagtttatg |
| pFusANTR-GFP | FusANTRfor | GAGCTCGAGATGCAGCAAAATGATACCTCTG |
|  | FusANTRrev | CACGGATCCCATCGACTGACTAGTCGGGG |
| pFusA-E49P | FusAE49Pfor | GTGATTCCTCTTCGCCGAACGGCGAAGATAC |
|  | FusAE49Prev | GTATCTTCGCCGTTCGGCGAAGAGGAATCAC |
| pFusA-E52P | FusAE52Pfor | CTTCGGAAAACGGCCCGGATACGATTTTAGTC |
|  | FusAE52Prev | GACTAAAATCGTATCCGGGCCGTTTTCCGAAG |
