# Supplementary material for: FusB Energizes Import across the Outer Membrane through Direct Interaction with Its Ferredoxin Substrate
Source: mBio. 2020 Oct 27;11(5):e02081-20. doi: 10.1128/mBio.02081-20 (PMC7593965; doi:10.1128/mBio.02081-20)
Supplement: TABLE S2 [file mBio.02081-20-st002.docx]

**Table S2. List of plasmids used in this study**

| Plasmid name | Vector backbone | Source |
| --- | --- | --- |
| pKD4 | pANTSγ | Datsenko & Wanner 2000 |
| pKD46 | pINT-ts | Datsenko & Wanner 2000 |
| FusA_NTR_-GFP | pWaldo (Waldo et al 1999) | This study |
| pFusA | pJ404 | This study |
| pFusB | pJ404 | This study |
| pFusC | pJ404 | Mosbahi et al 2018 |
| pFusB-CTD | pJ404 | This study |
| pTonB-CTD | pJ404 | This study |
| pExbB | pJ404 | This study |
| pFerAra | pET21 | Grinter et al 2016 |
| pFerPot | pET21 | Grinter et al 2016 |
